# SIRT1: A Potential Therapeutic Target in Autoimmune Diseases

**DOI:** 10.3389/fimmu.2021.779177

**Published:** 2021-11-23

**Authors:** Pan Shen, Xuan Deng, Zhe Chen, Xin Ba, Kai Qin, Ying Huang, Yao Huang, Tingting Li, Jiahui Yan, Shenghao Tu

**Affiliations:** ^1^ Department of Integrated Traditional Chinese and Western Medicine, Tongji Hospital, Tongji Medical College of Huazhong University of Science and Technology, Wuhan, China; ^2^ Department of Nephrology, Zhongnan Hospital of Wuhan University, Wuhan, China

**Keywords:** autoimmune diseases, SIRT1, inflammation, rheumatoid arthritis, systemic lupus erythematosus

## Abstract

The morbidity and mortality of autoimmune diseases (Ads) have been increasing worldwide, and the identification of novel therapeutic strategies for prevention and treatment is urgently needed. Sirtuin 1 (SIRT1), a member of the class III family of nicotinamide adenine dinucleotide (NAD^+^)-dependent histone deacetylases, has been reported to participate in the progression of several diseases. SIRT1 also regulates inflammation, oxidative stress, mitochondrial function, immune responses, cellular differentiation, proliferation and metabolism, and its altered functions are likely involved in Ads. Several inhibitors and activators have been shown to affect the development of Ads. SIRT1 may represent a novel therapeutic target in these diseases, and small molecules or natural products that modulate the functions of SIRT1 are potential therapeutic agents. In the present review, we summarize current studies of the biological functions of SIRT1 and its role in the pathogenesis and treatment of Ads.

## Introduction

Autoimmune diseases (Ads) are characterized by the dysregulation of the immune system, which results in the overproduction of autoantibodies, an imbalance in tolerance to self-antigens, and immune-mediated end-organ damage (1). Multiple types of Ads seriously affect the quality of life and labour ability and impose a substantial economic and psychological burden on society and families. To date, the etiology and pathogenesis of these diseases have remained invariably unknown, and complex and diverse elements associated with the environment, genetic risk factors, mental factors, and infection, which can cause an imbalance in autoimmune processes and immunological tolerance (2–4).

Sirtuin 1 (SIRT1), a nicotinamide adenine dinucleotide (NAD^+^)-dependent histone deacetylase, has been reported to participate in regulating various biological processes, such as energetic homeostasis (5), inflammation, oxidative stress (6), mitochondrial biogenesis (7), cell apoptosis (8), and autophagy (9). There have been preclinical and clinical studies indicating the significance of SIRT1 in the pathogenesis of Ads, including rheumatoid arthritis (RA), systemic lupus erythematosus (SLE), inflammatory bowel disease (IBD), multiple sclerosis (MS), etc. In women Hashimoto’s disease patients with type 1 diabetes mellitus, SIRT1 contributed to the pathogenesis of early cardiac dysfunction (10). Agathe et al. performed microarray experiments to identify SIRT1 as a relevant gene candidate associated with pathological angiogenesis in autoimmune arthritis mice (11). The deletion of SIRT1 in endothelial promoted a proliferative, proapoptotic and activated state of endothelial cells through the acetylation of p53 and p65, and resulted in the progress of proangiogenic capacities. The deficiency of SIRT1 in endothelial cells delayed the resolution of experimental arthritis. DNA hypomethylation was the first epigenetic pattern determined in SLE patients. The overexpression of SIRT1 was found in CD4+ T cells of a murine lupus model (12), however, SIRT1-null mice showed with immunoglobulin deposition in the kidneys and a high level of serum antinuclear antibody (13). The activator of SIRT1 plays a protective role in pristane-induced lupus mice, with the alleviation of proteinuria and decreased deposition of immune globulin in the kidneys (14). SIRT1 also may represent a biomarker of relapses and a potential target for therapeutic intervention in MS (15). The decreased expression of SIRT 1 has been found to increase the levels of proinflammatory cytokines that are being involved in the pathogenesis of IBD. On the contrary, the reduced expression of SIRT1 1 maintains the gastrointestinal barrier in IBD (16).

SIRT1 is a promising candidate molecule in the treatment due to different physiological and pathological processes in Ads. In the present manuscript, we will summarize and discuss the functions of SIRT1 in Ads and the therapeutic potential of targeting SIRT1 (**Table 1**).

**Table 1:** The role of SIRT1 modulation on autoimmune diseases.

Diseases	Animal models	Tissue/Cell types	Approaches	Main mechanisms and effects	References
SLE	MRL/*lpr* mice	Kidney/ splenic CD4+ T cells	SIRT1-siRNA	SIRT1 expression was suppressed and global histone H3 and H4 acetylation levels were elevated transiently in CD4+ T cells. Serum anti-dsDNA antibody level, renal IgG deposition, and renal pathological scores, tubulointerstitial scores, decreased significantly.	(17)
	Pristane induced lupus BALB/c mice model	Kidney/ splenic CD4+ T cells and CD19+ B cells	Resveratrol	Proteinuria, immunoglobuin depositon in kidney, and glomerulonephritis as well as IgG1 and IgG2a in serum decreased. CD4 IFNγ+ Th1 cells and the ratio of Th1/Th2 decreased. CD69 and CD71 expression on CD4+ T cells as well as CD4+ T cell proliferation was inhibited, CD4+ T cell apoptosis increased.	(18)
	SIRT1-null mice	Kidney	SIRT1 knockout	The immunoglobulin in the kidney was concentrated in the glomeruli. The frequency of anti-nuclear antibody was much higher.	(19)
RA	—	RA-FLSs	Silence of SIRT1	FLSs proliferation and leukocytic adhesion to FLSs reduced.	(20)
	—	RA-FLSs	Overexpression of SIRT1	FLSs proliferation, migration, and invasion was inhibited. RA-FLS apoptosis and caspase-3/8 activity increased.	(21)
	—	Peripheral blood monocytes/ RA-FLSs	Knockdown of SIRT1	Apoptosis of FLSs increased. IL-6 and IL-8 in FLSs reduced. Lipopolysaccharide-induced levels of TNFα in monocytes reduced.	(22)
	—	RA-FLSs	Resveratrol	Phosphorylation and acetylation of p65, c-Jun, and Fos was inhibited, and expression of COX-2 reduced.	(23)
	CIA mice	T cells	Resveratrol	The incidence and severity of CIA reduced. The translocation of c-Jun into the nucleus upon T cell activation was inhibited	(24)
	CIA rats	Synovial tissue/ rat synovial cells	Resveratrol	Levels of cell apoptosis were enhanced. Cell proliferation was inhibited. MAPK signaling, ROS accumulation, HIF-1α-mediated angiogenesis was inhibited.	(25)
	K/BxN serum transfer arthritis	Ankle tissue/ BMMs	mSIRT1 KO	IL-1, TNF-α, TRAP-positive osteoclasts, and F4/80+ macrophages in the ankles increased. Hyperacetylated p65 and increased NF-κB binding activity in BMMs, with increased M1 polarization, migration, pro-inflammatory cytokine production, and osteoclastogenesis.	(26)
	CIA mice	T cells and DCs	mSIRT1 KO	The mSIRT1 KO mice exhibited less severe arthritis, which was less destructive to the joints. ROR-γT, Th1 and Th17 cells, and CD80- or CD86-positive DCs reduced. The DCs showed decreases in T-cell proliferation and the Th1/Th17 immune response.	(27)
IBD	DSS-induced colitis	Colons	Resveratrol	TNF-α, IL-6, IL-1β, IFN-γ and IL-17 increased. The expression of TIMP-3 increased and TACE was inhibited.	(28)
	DSS-induced colitis	Colons/ macrophages and Caco-2 cells	SRT1720	Disease activity index, histological score, inflammatory cytokine levels, and apoptotic cell rate in colon tissues decreased. Levels of occludin and ZO-1 increased. The expression of GRP78, CHOP, cleaved caspase-12, cleaved caspase-9, and cleaved caspase-3 in Caco-2 cells and the colon tissues reduced.	(29)
	Radiation-induced inflammatory bowel disease	Intestine tissues	Resveratrol	The level of bowel inflammation reduced. The activity of NLRP-3 inflammasome was inhibited.	(30)
	C57BL/6* IL-10* deficient mice	Colon tissues	None	The levels of TNF-α increased and expression of SIRT1 decreased. The activation of the autophagy in mice from all stages.	(31)
	Chemically-induced colitis (TNBS or oxazolone)	Inflamed IBD mucosa	Cay10591	The activation of NF-κB and inflammatory cytokine synthesis was inhibited.	(32)
	DSS-induced colitis	T cells from spleens and lymph nodes	EX-527	Weight loss and increased iTreg formation.	(33)
MS	EAE mice	Optic nerve and RGC	Sirtinol/ SRT501	SIRT1 activators, SRT501, significantly attenuated RGC loss in a dose-dependent manner. This neuroprotective effect was blocked by sirtinol.	(34)
	MHV-A59 induced MS.	Optic nerve and RGC	SRTAW04	SIRT1 activating compounds prevent neuronal loss in viral-induced demyelinating disease involves increasing mitochondrial biogenesis with reduction of oxidative stress.	(35)
	EAE mice	Optic nerve and RGC	SRT501	Oral SRT501 prevented neuronal loss and neurological dysfunction during optic neuritis, an inflammatory optic nerve lesion in EAE.	(36)
	EAE mice	Optic nerve and RGC	Resveratrol	Resveratrol prevented neuronal loss in this chronic demyelinating disease model.	(37)
	EAE mice	Cerebellar tissue/ OPCs	Ex527	SIRT1 inhibition may help to expand the endogenous pool of OPCs without affecting their differentiation.	(38)
	EAE mice	Retina and optic nerves	Overexpression of SIRT1 within RGCs	SIRT1 mediated significant preservation of the OKR. SIRT1 gene augmentation was not able to suppress optic nerve inflammation or demyelination.	(39)
	EAE mice	Mononuclear cells from spleen and brain, and peritoneal macrophages	Resveratrol	EAE symptoms were significantly alleviated. Resveratrol protection against EAE is not associated with declines in IL-17+ T cells but is associated with rises in IL-17+/IL-10+ T cells and CD4-IFN-γ+ and with repressed macrophage IL-6 and IL-12/23 p40 expression.	(34)
	EAE mice	Spinal cords	Overexpression of SIRT1	SIRT1 activator suppressed EAE clinical symptoms and prevented or altered the phenotype of inflammation in spinal cords. Demyelination and axonal injury were reduced.	(35)
	EAE mice		NAD+	NAD+ treatment could lessen the severity of EAE and suppress pro-inflammatory T cell responses. SIRT1 pathway was activated in the NAD+-treated.	(36)

ROS, reactive oxygen species; HIF-1α, hypoxia-inducible factor-1α; MAPK, mitogen-activated protein kinase; NF-κB, nuclear factor-κB; mSIRT1 KO, myeloid cell-specific SIRT1 knockout; CIA, collagen-induced arthritis; DCs, dendritic cells; DSS, dextran sulfate sodium; TACE, TNF-α converting enzyme; TIMP-3, Tissue inhibitor of metalloproteinase-3; BMMs, bone marrow-derived monocytes/macrophages; ZO-1, zona occludens 1; GRP78, glucose-regulated protein 78; CHOP, CCAAT/enhancer-binding protein homologous protein; TNBS, 2,4,6-trinitrobenzenesulphonic acid; EAE, experimental autoimmune encephalomyelitis; RGC, retinal ganglion cell; OPC, oligodendrocyte progenitor cell; OKR, optokinetic response; NAD+, nicotinamide adenine dinucleotide.

## Sirtuins and SIRT1

Acetylation, an evolutionarily conserved posttranslational modification of lysine residues, mainly facilitates chromatin formation and gene transcription. Protein deacetylases eliminate the effect of protein acetyltransferases by removing the acetyl groups added to the lysine residues (40). These enzymes are called histone deacetylases (HDACs), which are divided into four classes in mammals (41). Class III HDACs or silent information regulator 2 (Sir2) are NAD^+^-dependent HDACs that modify histone proteins and nonhistone proteins *via* deacetylation (42). Sirtuins, a family of highly conserved NAD^+^-dependent HDACs, share homology with Sir2 of the yeast *Saccharomyces cerevisiae* and show no sequence similarity to the other HDACs (43). According to existing studies, humans have 7 sirtuin paralogues, SIRT1–7, characterized by different binding targets, tissue specificities, functions, and localization (44, 45). SIRT1, SIRT6, and SIRT7 are mainly distributed in the nucleus (46, 47). SIRT2 is mainly found in the cytoplasm. SIRT1 and SIRT2 also share nuclear-cytoplasmic shuttling (48). SIRT3, SIRT4, and SIRT5 are located in mitochondria, and SIRT3 is also expressed in the nucleus under normal conditions (49–51). SIRT1, the protein with the largest molecular mass (120 kDa) and member of the sirtuin family with the highest amino acid sequence homology with yeast SIR2 (52), has been most widely studied (53).

*SIRT1*, located on chromosome 10q21.3, consists of 8 introns and 11 exons. The structure of the SIRT1 protein contains 747 amino acid residues and a catalytic core region flanked by variable NH_2_- and COOH-terminal domains consisting of approximately 250 amino acids (54). The variety of terminal domains is associated with the diversity of sirtuin functions. This domain forms the hairpin structure that compliments with the β sheet of the NAD^+^-binding domain, and the NH_2_-terminal domain potentiates the catalytic activity (55). In addition, extensions of the NH_2_- and COOH-termini influence the functions of SIRT1, which are the targets of posttranslational modifications (56). Through the action of NAD^+^, SIRT1 removes the acetyl moieties of ϵ-acetyl-lysine residues of histones and other target proteins, thereby producing 2’-O-acetyl-ADP-ribose, nicotinamide, and the deacetylated substrate (57, 58). SIRT1 not only deacetylates lysine residues of histones, such as lysine 16 of H4, lysine 26 of H1, and lysine 9 of H3, but also regulates the activity of a number of transcription factors *via* deacetylation (59). SIRT1 epigenetically silences these target proteins at the transcriptional or posttranslational level, such as forkhead box class O (FoxOs), p53, nuclear factor-κB (NF-κB), nuclear factor E2-related factor 2 (Nrf2), HIF1α, AMP-activated protein kinase (AMPK), β-catenin, mitochondrial peroxisome proliferator-activated receptor γ coactivator 1 alpha (PCG-1α), proliferator-activated receptor gamma (PPARγ), and Notch. SIRT1 participates in a series of pathological and physiological processes, including cell metabolism and DNA repair. The regulation of diverse physiological signalling pathways and targets by SIRT1 makes it a promising therapeutic target. However, the specific mechanism of SIRT1 in Ads is unknown.

## Immunological Functions of SIRT1

Due to continuous exposure to a variety of pathogenic agents, such as bacteria, viruses, and fungi, the body defends against these potentially fatal infections through a series of highly regulated responses known as innate and adaptive immunity. The targets of SIRT1 can affect immune cells and immune responses to modulate the progression of chronic autoimmune and inflammatory diseases (**Table 2**).

**Table 2:** The Immunological Functions of SIRT1.

Immune cell types	Treatment	Activity of SIRT1	Targets	Effects	References
Macrophages	Knockout of SIRT1	Deletion	NF-κB p65	The expression of TNF-α, IL-1β increased.	(60)
	Resveratrol	Elevated	JNK and IKK	The expression of TNF-α decreased.	(61)
	Nicotinamide	Inhibition	E2F1, Myc, FoxO1	Cell cycle progression and renewal was inhibited.	(62)
	siRNA	Inhibition	c-Fos, c-Jun	The expression of COX2 and PGE2 decreased.	(63)
Dendritic cells	EX-527	Inhibition	HIF1α	The expression of IL-12 increased and TGFβ-1 decreased.	(64)
	Cambinol/ sirtinol	Inhibition	PPARγ, Th2	Allergic inflammation was inhibited.	(65)
	Knockout of SIRT1	Deletion	IRF1	IL-27 production and Th17 differentiation was suppressed.	(66)
	EX-527/ knockout of SIRT1	Inhibition/ deletion	–	Th2 cytokine production enhanced *in vivo* with EX-527. Inflammatory cytokine gene expression and autophagy was attenuated in vitro with EX-527 and *in vivo* with SIRT-null.	(67)
CD4+ T cells	Knockout of SIRT1	Deletion	Bclaf1	The levels of IL-2 increased and T cell apoptosis enhanced.	(68)
	EX-527	Inhibition	RORγt	The differentiation of Th17 cells and production of IL-17 was inhibited.	(69)
	Resveratrol/ SRT720	Elevated	STAT3	Th17 differentiation was inhibited.	(70)
	Knockout of SIRT1	Deletion	mTOR, HIF1α	Th9 cell differentiation was promoted and IL-9 levels increased.	(71)
	EX-527	Inhibition	FoxP3	The differentiation and stability of Tregs was enhanced.	(72)
B cells	Knockout of SIRT1	Deletion	NF-κB p65, DNMT1	Increased AICDA levels, and the induction of antibody maturation.	(73)

FOXOs, factor forkhead box protein Os; COX2, cyclooxygenase 2; PGE2, prostaglandin E2; TGF-β, transforming growth factorβ; FOXP3, factor fork head box P3; IRF1, interferon regulatory factor 1; RORγ t, retinoid acid receptor-related orphan receptor gamma t; Bclaf1, B-cell lymphoma 2-associated factor 1; DNMT1, DNA methyltransferase 1; AICDA, activation-induced cytidine deaminase.

## Innate Immunity

Circulating monocytes from the blood enter the tissue and then develop into macrophages after differentiation and maturation. These phagocytes affect the immunopathogenesis of Ads. In SIRT1-deficient macrophages, hyperacetylation of NF-κB p65 results in increased levels of proinflammatory cytokines, such as tumour necrosis factor (TNF)-α and interleukin (IL)-1β, compared to the control (60). Furthermore, mice lacking SIRT1 in macrophages have high levels of activated macrophages in the liver and adipose tissues, which promote insulin resistance and metabolic syndrome. Clearly, p50/p65 is located in the cytoplasm mainly through an interaction with the inhibitor protein κB (IκB), and the activation of macrophages causes the degeneration of IκB, transferring NF-kB to the nucleus and finally promoting the expression of inflammatory genes (74). In RAW264.7 macrophages, siRNA-mediated knockdown of SIRT1 increased the expression of activated NF-κB and inflammatory factors and cytokines (61). Imperatore et al. reported an essential role for SIRT1 in the self-renewal of macrophages through the regulation of the cell cycle and longevity pathways (62). Overexpression of SIRT1 in bone marrow-derived macrophages increases their proliferative capacity. Silencing and deleting the SIRT1 gene restricts the self-renewal of macrophages. Moreover, SIRT1 inhibition negatively regulates the G1/S transition, cell cycle progression, and renewal, which are associated with the inhibition of E2F1 and Myc and the activation of FoxO1. Activator protein-1 (AP-1), which is composed of c-Fos and c-Jun, is also regulated by SIRT1 to affect inflammatory gene transcription. SIRT1 binds to c-Fos and c-Jun and deacetylates c-Jun, thereby inhibiting the transcriptional activity of AP-1 in peritoneal macrophages and reducing AP-1-associated expression of inflammatory mediators, including cyclooxygenase-2 (COX2) and prostaglandin E2 (63).

Dendritic cells (DCs) play a key protective role in promoting antigen-specific responses by adaptive immune cells and in producing a variety of chemokines and cytokines that recruit immune cells into the target tissues following the invasion of pathogenic microorganisms (75). SIRT1 regulates the generation of cytokines, such as IL-12 and TGFβ-1, by DCs through HIF1α modulation, which potentially regulates the formation of helper T (Th)-1 cells and regulatory T cells (Tregs) and the function of DCs (64, 76). The inhibition of SIRT1 in DCs promotes the differentiation of Th1 cells while restricting the differentiation of Tregs. In a mouse colitis model, transferring naïve CD4^+^ T cells into mice with DCs with a specific deletion of *SIRT1* aggravated colonic inflammation and promoted weight loss. In cocultures of activated DCs with CD4^+^ T cells, pharmacological SIRT1 inhibition increases the Th1/Treg ratio and the expression of IFN-γ and IL-12 and reduces the expression of TGFβ1 (77). In a mouse asthma model, pharmacological inhibition of SIRT1 increases the activity of PPARγ and inhibits Th2 cell responses in allergic airway inflammation, which are associated with an imbalance in the maturation and migration of lung DCs (65). The loss of SIRT1 limits DC transfer into the draining lymph nodes, leading to disrupted Th2 cell differentiation. In a mouse OVA-induced airway inflammation model with DC-specific deletion of SIRT1 (66), the maturation and migration of DCs are reduced. Coculture of activated SIRT1-deficient DCs with CD4^+^ T cells inhibits the differentiation of Th17 cells, which is reversed by anti-IL-27 and anti-IFN-β antibodies. In respiratory syncytial virus infection, SIRT1 promotes the activation of DCs to produce efficient antiviral immune responses (78).

## Adaptive Immunity

The role of SIRT1 in the adaptive immune response was mainly identified to be a negative regulator of T cell function. The levels of SIRT1 are increased in activated T cells and anergic T cells compared to mature naïve T cells (67). Compared with wild-type littermates, CD4^+^ T cells from SIRT1-deficient mice appear to exhibit greater proliferation and cytokine production (67). However, mice with a SIRT1 deletion do not present abnormal T or B cells, suggesting that SIRT1 is unlikely to be a key factor contributing to the activation of T or B cells. SIRT1 deficiency is still an important factor associated with a higher risk of Ads (79).

The hyperactivation of T cells with a specific deletion of SIRT1 is likely to be associated with the loss of inhibition of NF-κB and AP-1 activity. SIRT1 inhibits the AP-1 signalling pathway mainly through the deacetylation of c-Jun, thereby inhibiting T cell activation and proliferation. In addition to directly regulating the activity of transcription factors, SIRT1 regulates related genes that affect T cell proliferation and function. Bclaf1, initially identified as a Bcl-2-binding protein, is considered required for T cell activation (80). SIRT1 is a suppressor of Bclaf1 transcription that inhibits the activity of NF-κB and deacetylates histone lysine residues. The differences in Bclaf1 levels and Bclaf1 locus histone deacetylation between wild-type animals and mice with systemic knockout of SIRT1 are not significant in naïve, un-activated T cells (81). In the presence of IL-6, IL-23, and TGFβ, naïve T cells cultured with antigen-presenting cells induce the generation of Th17 cells, which have been proven to participate in the immunopathogenesis of certain Ads (68). SIRT1 is expressed at high levels in Th17 cells and plays an essential role in Th17 cell formation. SIRT1 binds to and deacetylates the transcription factor retinoid acid receptor-related orphan receptor gamma (RORγt), promoting the differentiation of Th17 cells by activating IL-17 and repressing the IL-2 promoter (82). Mice with a targeted deletion of SIRT1 in T cells showed a reduction in Th17 differentiation through the suppression of IL-17 expression and induction of IL-2 expression. Additionally, SIRT1 also limits Th17 differentiation by deacetylating signal transducer and activator of transcription (STAT)-3, which is required for RORγt transcription (69). Several studies found that increasing rather than suppressing SIRT1 may inhibit Th17 differentiation (70, 83). More evidence is needed to prove the direct effect of SIRT1 on Th17 development. SIRT1 also functions as a negative regulator in the differentiation of IL-9-secreting effector cells and Th9 effector cells, which have been shown to possess antitumour and antiallergic activities (84). Targeted deletion of SIRT1 in mouse CD4^+^ T cells or silencing of SIRT1 in mouse or human T cells promotes the differentiation of Th9 cells and IL-9 production, whereas ectopic expression of SIRT1 inhibits IL-9 production and Th9 differentiation (85). Additionally, IL-9 produced by SIRT1-deficient T cells protects against tumours and increases the levels of allergic pulmonary inflammation.

IL-2 plays an important role in the proliferation of activated T cells and in preventing their apoptosis induced by the high-affinity IL-2 receptor CD25 through the phosphoinositide-3- kinase (PI3K)/Akt pathway to suppress FoxO1, 3, and 4 (71). SIRT1 is a very important deacetylase of FoxO family members. Normally, SIRT1 is likely to regulate the activation of T cells *via* the deacetylation of FoxO proteins and the inhibition of FoxO apoptotic signalling and the IL-2 signalling pathway.

SIRT1 regulates the acetylation and stability of FoxP3, a crucial transcription factor involved in the differentiation of Tregs (86). Hyperacetylation of FoxP3 diminishes its polyubiquitination and increases its stability. The inhibition of sirtuins downregulates the acetylation of FoxP3 and promotes the ubiquitination and degeneration of FoxP3. In addition, the Notch receptor in Tregs plays an essential role in the survival of Tregs and is associated with the antiapoptotic effect of the Notch1 intracellular domain. SIRT1 was shown to stabilize the Notch1 intracellular domain proximal to the membrane to promote Treg survival (87). However, in contrast, SIRT1 inhibition promotes the formation of FoxP3^+^ Tregs with elevated immunosuppressive activity (88). Beier et al. reported that conventional CD4^+^FoxP3^-^ T cell-specific deletion of SIRT1 in mice rarely affected the numbers of T cells and their activation and proliferation but increased the expression of FoxP3 and suppressive activity of Tregs *in vitro* and *in vivo* (72). Compared to wild-type mice, mice with specific deletions of SIRT1 in FoxP3^+^ Tregs survived significantly longer with mismatched heart allografts. Wild-type mice treated with SIRT1 selective inhibitors (splitomicin and EX-527) showed similar results. Another study reported that mice with a SIRT1 deletion and mismatched renal allografts experienced longer survival than wild-type mice (89). CD4^+^ T cells isolated from a cervical heterotopic heart transplantation mouse model treated with sirtinol (sirtuin inhibitor) showed significantly lower expression of IL-17A and RORγt and higher expression of FoxP3 (90). *In vivo*, sirtinol reduces the differentiation of Th17 cells and increases the proportion of Treg cells among splenocytes. Additionally, co-transfection of SIRT1 with FoxP3 increases FoxP3 proteasomal degeneration, while SIRT1 inhibition increases FOXP3 transcriptional activity in human Treg (91). SIRT1 inhibition might increase FoxP3 acetylation to promote the production and functions of FoxP3^+^ Tregs and inhibit the acetylation of RORγt and the differentiation of Th17 cells, thereby affecting the Th17/Treg ratio (92, 93). Nonetheless, *SIRT1^-/-^* mice develop spontaneous and severe Ads (67) but not mice with SIRT1-deficient CD4^+^ T cells. A potential explanation for this finding is alterations in thymic T cell selection in *SIRT1^-/-^* mice and the expression of autoimmune regulator (AIRE), which is required for the former. SIRT1 also plays a key role in regulating the expression of AIRE (79).

The function of SIRT1 in another group of lymphocytes has rarely been studied. SIRT1 regulates the activation of B cells through CD38 and NAD (94). In another study, SIRT1 regulated antibody maturation in B cells (95). The activation of B cells resulted in the inhibition of SIRT1 and the upregulation of activation-induced cytidine deaminase (AICDA). B cells obtained from mice with B cell-specific deletion of SIRT1 showed reduced deacetylation in activated B cells, increased AICDA levels, and the induction of antibody maturation. More studies are needed to clarify the role of SIRT1 in the activation and differentiation of B cells.

## SIRT1 in Autoimmune Diseases

### SLE

SLE is a multisystemic and chronic inflammatory disorder characterized by autoantibody production, immune complex deposition, inflammation, and damage to multiple tissues and organs. Levels of the SIRT1 mRNA and protein are significantly increased in patients with active lupus nephritis (LN) compared with those in remission or healthy patients (73). Moreover, histological features of LN biopsies were related to increased SIRT1 expression in proliferative forms. SIRT1 expression showed a strong power to discriminate kidney damage in patients with SLE. Hu et al. found that the administration of SIRT1-siRNA to MRL/*lpr* mice significantly increases levels of acetylated H3 and H4 in CD4^+^ T cells and reduces serum anti-dsDNA antibody levels and renal pathological scores, particularly tubulointerstitial scores (12). The results suggested that the overexpression of SIRT1 *in vivo* was associated with lupus pathogenesis and that SIRT1 inhibition mitigated the damage induced by lupus in MRL/*lpr* mice. Consiglio et al. analysed genomic DNA from the peripheral blood of 367 patients with SLE and 290 healthy controls in a Brazilian population and found that SIRT1 promoter variant rs3758391 modifies SLE morbidity, with the rs3758391 T allele serving as a risk factor for nephritis and a higher systemic lupus erythematosus disease activity index (SLEDAI) (96). Nevertheless, researchers have not yet elucidated how the SIRT1 rs3758391 variant functionally affects SLE severity. In another study, the activity of DNA methyltransferase 1 (DNMT1) was inhibited in CD4^+^ T cells isolated from 22 patients with active SLE transfected with si-SIRT1. Ultraviolet B radiation suppressed SIRT1 mRNA and protein expression by activating aryl hydrocarbon receptor (AhR) and downregulated the activity of DNMT1 in CD4^+^ T cells by binding to the SIRT1 promoter (97). B cell hyperactivity is a major characteristic of SLE and is involved in the progression of SLE. Wang et al. (17) transfected mouse B cells BaF3 with a SIRT1 vector or shRNA targeting SIRT1, and the results showed that SIRT1 overexpression promotes BaF3 cell proliferation and increases the expression of proinflammatory cytokines (IL-6 and TNF-α). In addition, p65 was significantly activated and phosphorylated, and the expression of B cell CLL/lymphoma 3 (Bcl-3) was increased. SIRT1 might be a potential risk factor for the development of SLE.

**Figure 1:**
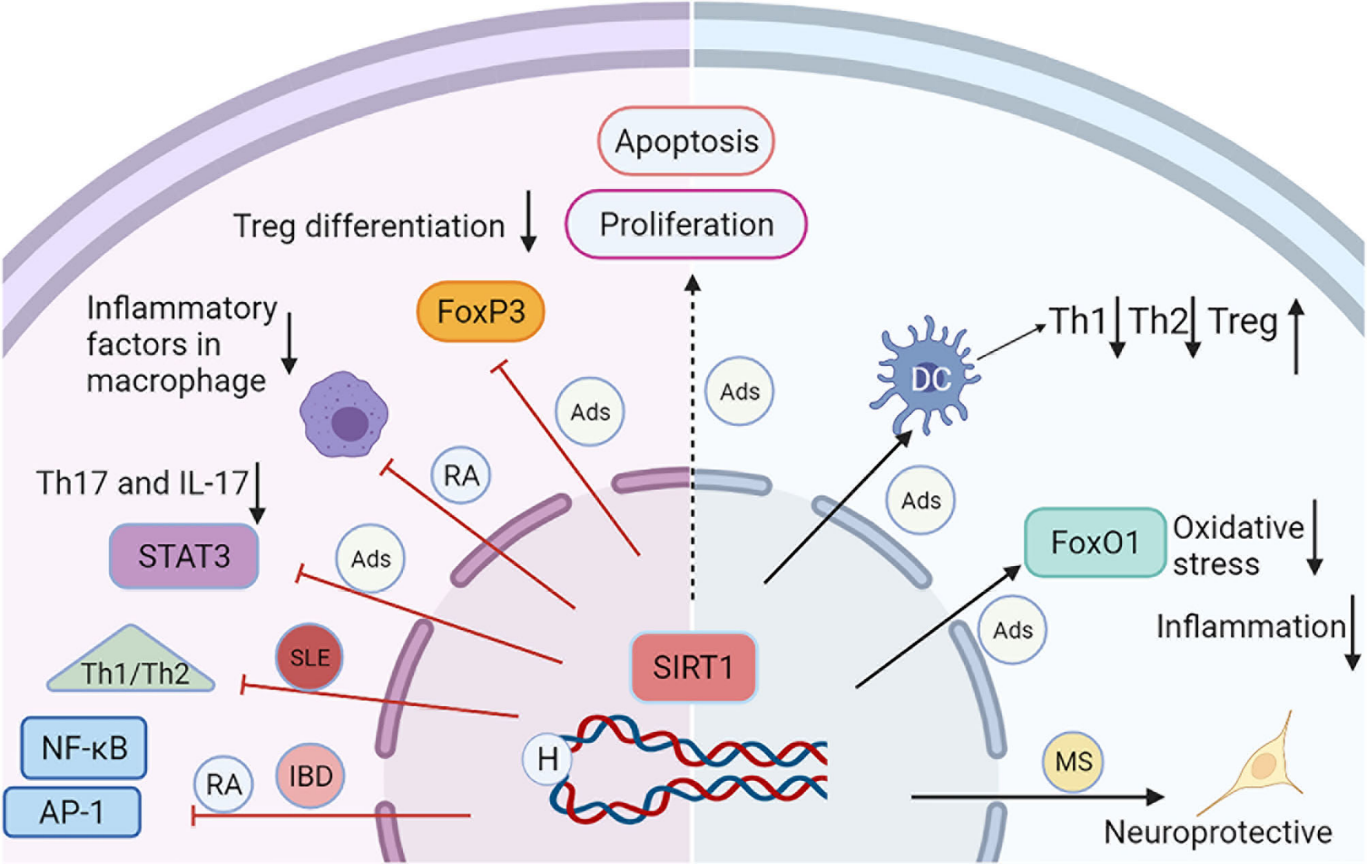
A schematic diagram illustrating the major mechanisms of SIRT1 in selected autoimmune diseases. The activation of SIRT1 reduces the acetylation and activation of transcription factors, such as NF-kB, STAT3, AP-1, and FoxP3, leading to decreased inflammation, and apoptosis. SIRT1 can inhibit inflammation and oxidative stress through the deacetylation of FoxO1. SIRT1 also regulates DC-mediated Th1, Th2, and Treg immune responses. All these processes interact each other and contribute to the progression of Ads.

In contrast, the activation of SIRT1 by resveratrol, a SIRT1 activator, attenuates proteinuria, glomerulonephritis, and the serum levels of IgG1 and IgG2a in mice with pristane-induced lupus (14). Furthermore, resveratrol also suppresses CD69 and CD71 expression on CD4^+^ T cells, as well as CD4^+^ T cell proliferation, induced CD4^+^ T cell apoptosis, and decreased the number of CD4 IFNγ^+^ Th1 cells, the proliferation of B cells, and the ratio of Th1/Th2 cells *in vitro* (as shown in **Figure 1**). Resveratrol protects against lupus-induced tissue damage and may represent a potential therapeutic agent for the treatment of SLE. Moreover, SIRT1 knockout mice show higher levels of anti-dsDNA and anti-nuclear antigen IgG and IgM immunoglobulin than wild-type mice (13). A potential explanation is that histone modification dysregulation is related to the incidence of SLE (98). The deletion of SIRT1 in activated B cells results in the production of autoantibodies targeting nuclear antigens, dsDNA, and ribonucleoprotein (95). Compared to healthy controls, B cells obtained from mice and patients with SLE exhibit increased expression of AICDA, which is related to decreased SIRT1 expression. However, the occurrence of SLE is also related to the increase in SIRT1 levels, which may be due to the overall imbalance of transcription and hyperacetylation.

### RA

RA is one of the most prevalent chronic disorders defined as a systemic breakdown of self-tolerance and immune-mediated inflammation characterized by cartilage/bone destruction and joint dysfunction (99). The inflammatory proliferation of RA fibroblast-like synoviocytes (FLSs) is the most important pathological feature of RA, which generates invasive synovial pannus and a series of pro-inflammatory cytokines and produces proteases that directly destroy bone and cartilage. Therefore, an effective method to slow the progression of RA is to promote apoptosis and inhibit the proliferation, invasion, and migration of FLSs, which suppress synovial inflammation and alleviate joint deformation. SIRT1 activation induces the apoptosis of FLSs through the activation of caspase-3 and the PI3K/Akt signalling pathway. Engler et al. found that SIRT1 silencing promotes the proliferation and adhesion of FLSs (100). SIRT1 overexpression not only reduces the production of proinflammatory cytokines but also inhibits the proliferation, invasion, and migration of FLSs, thereby effectively alleviating RA synovial inflammation (18, 19). These effects are associated with the activation of NF-κB (as shown in **Figure 1**). The upregulation of SIRT1 reduces COX2 levels in RA-FLSs by inhibiting the activation of AP-1 and NF-κB (101). The DNA binding activity of AP-1 is significantly increased in the synovial tissues of patients with RA and positively correlates with the disease activity of RA. Inhibition of AP-1 activity helps alleviate the disease (102).

SIRT1 activation by resveratrol reduces the activation of T cells, the production of TNF-α and IL-12, and the expression of CD28 and CD80 and upregulates CTLA4 expression in collagen-induced arthritis (CIA) mice and T cells. SIRT1 inhibition in T cells suppresses the resveratrol-induced inhibition of T cells and increases the acetylation of c-Jun and the incidence and severity of CIA (20). Resveratrol inhibits the activation of the MAPK signalling pathway and the expression of IL-1β in synovial tissues of CIA rats and exerts a positive regulatory effect on the development of arthritis (21). In mice with myeloid cell-specific deletion of SIRT1, an arthritis model (serum transfer from K/BxN arthritis mice) showed more severe inflammatory responses and pathological changes, including increases in IL-1β and TNF-α levels, as well as increases in the number of TRAP^+^ osteoclasts in the ankles (22). Compared to wild-type mice, macrophages obtained from mice with selective deletion of SRIT1 show increased migration, polarization, and proinflammatory cytokine production, which are associated with hyperacetylation of p65 and activation of NF-κB. Based on these results, SIRT1 suppresses the activation of innate immune cells, leading to less joint inflammation and damage. In contrast, Woo et al. (23) observed less joint inflammation and damage in myeloid cell-specific SIRT1 knockout arthritis mice than in wild-type mice with CIA. SIRT1 inhibition in CIA mice also reduces the production of inflammatory cytokines, MMPs and RORγt and decreases the proliferation of Th1, Th17, and DCs. In addition, impaired DC maturation and a reduction in the Th1/Th17 immune response are observed in these mice. The explanation for these differences among different studies may be attributed to the different pathogeneses in different arthritis models. The serum transfer model from K/BxN arthritis mice does not require the participation of T, B, and innate immune cells, which are required to produce autoantibodies in the CIA model.

Patients with RA suffer from multiple cartilage/bone erosion events and osteoporosis in the middle and late stages, a common clinical complication that can lead to joint deformity, severe dysfunction, and disability. SIRT1 heterozygous female mice show a significant decrease in bone density, suggesting that SIRT1 plays a role in regulating bone metabolism (103). Osteoblast-specific deletion of SIRT1 in mice significantly reduces osteoblast differentiation and bone mass and promotes the activation of the NF-κB signalling pathway and the differentiation and maturity of osteoclasts (24). Activated SIRT1 deacetylates NF-κB and p53 and reduces IL-1β, iNOS, and IL-6 levels and the inflammation and apoptosis of articular chondrocytes (25). Therefore, SIRT1 modulates the erosive destruction of articular cartilage and bone by regulating the differentiation, maturation, and apoptosis of osteoblasts, osteoclasts, and chondrocytes, reducing the disability rate of individuals with RA.

### IBD

IBD is characterized by chronic relapsing intestinal inflammation and gastrointestinal bleeding, and includes two main types: Crohn’s disease and ulcerative colitis. IBD has received increasing attention in recent decades due to its increasing incidence rate worldwide (more than 2 million individuals have been diagnosed with IBD), especially in China. Although the exact pathogenesis of IBD is not fully understood, genetics, intestinal microbiota, and environmental factors have been considered as main regulators. The identification of new targets and definitive methods for IBD treatment is urgently needed. Several studies have reported that decreases in SIRT1 expression are critical for the development of IBD.

Sharma et al. (26) used dextran sulfate sodium-induced (DSS) IBD mice, which are the most commonly used IBD model because they share many manifestations and pathological characteristics with human disease, to explore the role of SIRT1 in the development of colonic inflammation. Treatment with resveratrol significantly improves DSS-induced colitis and restores the SIRT1 mRNA levels. Ren et al. (27) found that the SIRT1 activator SRT1720 decreases the disease activity index, inflammatory cytokine levels, and colon histological score in mice with DSS-induced colitis, whereas nicotinamide (SIRT1 inhibitor) administration exerts the opposite effects. Resveratrol suppresses the activation of the NLRP-3 inflammasome and alleviates bowel inflammation in mice with radiation-induced IBD (104). SIRT1 also participates in the development of chronic spontaneous colitis in an IL-10-deficient mouse model (105). Talero et al. found that IL-10-deficient mice are characterized by increased levels of cytokines and decreased SIRT1 mRNA levels in the colonic mucosa, which are associated with the upregulation of the autophagy pathway, promoting inflammation and dysplasia in mice. Caruso et al. (106) detected reduced levels of the SIRT1 mRNA and protein in colon tissues from mice with 2,4,6-trinitrobenzenesulfonic acid-induced colitis. The administration of a SIRT1 antagonist, EX-527, to mice increased the disease severity and infiltration of CD3^+^ T cells in the colon. Cay10591, a SIRT1 agonist, decreased the generation of proinflammatory cytokines. Moreover, the expression of the SIRT1 mRNA and protein was decreased in lamina propria mononuclear cells from patients with IBD, while treatment with a SIRT1 activator inhibited the activation of NF-κB and the generation of proinflammatory cytokines. Taken together, SIRT1 activation attenuates colitis, and SIRT1 may represent a promising target for treating IBD.

In contrast, several studies also found that SIRT1 may stimulate the pathogenesis of IBD. In one study, intestinal-specific deletion of SIRT1 protected mice from the development of colitis (28). DSS did not induce colitis successfully in SIRT1-deficient mice with decreased expression of inflammatory genes. Thus, the deletion of SIRT1 in the intestine exerts a positive effect on the development of IBD. In another study, Akimova et al. primarily determined the role of SIRT1-targeted T cells in the development and pathogenesis of chronic colitis in mice (29). The authors reported that adoptive transfer of CD4^+^ CD25^-^ Foxp3^-^ T effector (TE) cells from wild-type mice into B6/Rag1^-/-^mice induced chronic colitis, which was related to the expansion of disease-producing Th1 effector cells that promoted increases in weight loss and the infiltration of T cells into the colon. Moreover, the adoptive transfer of TE cells from SIRT1-deficient mice into B6/Rag1^-/-^mice resulted in lower colitis disease activity and reduced weight loss, as well as a 2.8-fold increase in the formation of iTregs, compared with mice receiving wild-type T cells. Therefore, naïve T cells tend to differentiate into iTregs in the absence of SIRT1. In a second mouse model, treatment with a SIRT1 inhibitor, EX-527, reduced weight loss and colonic inflammation and increased iTreg differentiation. The deletion of SIRT1 may inhibit the development of colitis through the induction of Tregs.

Both protective and deleterious effects of SIRT1 have been reported on individuals with IBD. The protective actions of SIRT1 are associated with decreased acetylation of NF-κB, which results in increased expression of proinflammatory cytokines. In contrast, the deletion or silencing of SIRT1 inhibits colitis through the induction of Tregs, which are essential for the maintenance of gastrointestinal homeostasis.

### MS

MS is a chronic neuroinflammatory and demyelinating disease. Genetic and environmental factors may influence the susceptibility to and progression of MS. Several recent studies have provided evidence supporting the beneficial effects of SIRT1 on demyelinating and inflammatory diseases, such as MS. Compared with patients with MS who are in remission and healthy controls, SIRT1 activity is significantly decreased in patients with active MS (30). In addition, SIRT1 colocalizes with CD4^+^, CD68^+^, oligodendrocytes (OLGs), and glial fibrillary acidic protein-positive cells in MS lesions. Although a wide distribution of cells expresses SIRT1, higher SIRT1 expression was detected in the MS lesions than in the area adjacent to the MS lesions. Moreover, peripheral blood mononuclear cells (PBMCs) from patients with active MS expressed higher levels of the SIRT1 mRNA and protein than those from patients in remission and healthy controls. Based on these results, SIRT1 may represent a biomarker of relapse (15). Ciriello and Hewes et al. (31, 32) found that phosphorylated SIRT1 (p-SIRT1) and H3K9me3 are possible biomarkers for MS relapse, and SIRT1 and H3K9me3 potentially predict the response to glatiramer acetate (GA, a widely used drug in patients with MS) therapy. Higher SIRT1 mRNA and H3K9me2 levels are detected in responders to GA treatment than in nonresponders.

Shindler et al. tested whether activators of SIRT1, namely, SRT647 and SRT501, prevent neuronal loss caused by optic neuritis in an SJL/J model of experimental autoimmune encephalitis (EAE) (107). Activated SIRT1 inhibits retinal ganglion cell loss in a dose-dependent manner. In contrast, the inhibition of SIRT1 with sirtinol blocks the neuroprotective effects. In addition, activated SIRT1 increases the axonal density, protecting against neuronal damage and long-term neurological dysfunction. However, treatment with an activator does not reduce the disease index of EAE or attenuate optic nerve inflammation, indicating that neuroprotection is not associated with immunosuppression (as shown in **Figure 1**). In another study, SIRT1 activation attenuated optic neuritis induced by a neurotropic strain of hepatitis virus and MHV-A59 and reduced ROS levels (33). In addition, the activation of SIRT1 by oral resveratrol reduced disease severity in a mouse model of chronic EAE (108) and decreased neuronal loss and paralysis (109). SIRT1 protein expression was upregulated in the nuclei of NG2^+^ or PDGFRα^+^ oligodendrocyte progenitor cells in demyelinated brain lesions, which may inhibit the regeneration of functionally competent oligodendrocytes (110). McDougald et al. investigated the neuroprotective potential of SIRT1 using adeno-associated virus vector gene transfer in an EAE mouse model. Vector-SIRT1 improved the optokinetic response and protected retinal ganglion cells compared to Vector-eGFP controls (111). Treatment with resveratrol also reduces the production of proinflammatory cytokines, such as IL-6 and IL-12/23 p40, in EAE mice (34). SIRT1 overexpression in EAE mice significantly decreases the clinical score, inflammation, and myelin loss and improves axon preservation and neuronal survival (35). The neuroprotective effects appear to be associated with upregulated levels of NAD^+^ and brain-derived neurotrophic factors. Treatment with NAD^+^ for EAE in C57BL/6 mice alleviates the severity of EAE and activates SIRT1 (36). T cells are involved in regulating SIRT1 expression and the pathogenesis of most autoimmune syndromes, including MS. However, the regulatory effects of T cells on MS are not fully understood. Zhang et al. reported that adiponectin inhibits Th17 cell-mediated mouse autoimmune CNS inflammation. This process might be associated with increases in SIRT1 and PPARγ levels and the inhibition of RORγt and Th17 cell differentiation (37). Wang et al. reported that methylene blue reduces the clinical indices of mouse EAE models and attenuates pathological injuries in the spinal cord. The protective effects are associated with activation of SIRT1 and the Th17/Treg balance (38). SIRT1 inhibition increases the expression of FasL and promotes the apoptosis of CD4^+^ and CD8^+^ cells from patients with MS (39).

## Conclusions

In recent years, the function of SIRT1 has expanded far beyond its initial impression as a prominent NAD^+^-dependent class III HDAC of the sirtuin family. SIRT1 participates in the complex coordination of the immune system and Ads. Although various articles have examined the roles of SIRT1 in suppressing the promotion of autoimmune diseases, many studies described in this review support the hypothesis that SIRT1 represents a possible biomarker of relapses and a potential target for therapeutic intervention in multiple Ads, including SLE, RA, IBD, MS, regardless of whether it functions as an activator or inhibitor. SIRT1 regulates the expression and activity of some transcription factors and genes, affecting immune cell activation, differentiation, and function.

SIRT1 not only affects histones deacetylation, but also deacetylation of various transcription factors, including p65, p53, FoxO family, STAT3, PGC1α, and PPARγ, leading to transcription repression. SIRT1 regulates the activity of p53 and FoxO3 through deacetylation and promotes cell survival *via* suppression of apoptosis and cell death in response to DNA damage and oxidative stress. The deficiency of SIRT1-mediated deacetylation of FoxO3a causes increased etoposide-induced apoptosis. On bone tissues, SIRT1 maintains its “self-renewal” ability through the inhibition of inflammation, oxidative stress, and senescence. SIRT1 also exerts anti-inflammatory effects through the inhibition of NF-kB, AP-1, and STAT3 pathways. SIRT1 causes the deacetylation and inactivation of STAT3 during caloric restriction. SIRT1 inhibits the NF-kB pathway through deacetylation of p65 and regulates cellular response to hypoxia *via* deacetylation of HIF-1α. However, loss of SIRT1 might lead to improved immune surveillance against pathogenic infection and nonself antigens, not all diseases benefit from the activation of SIRT1 or might even worsen Th2-mediated immune responses. All these highlight an important transcription modulatory function by SIRT1 activity, and the essential roles of SIRT1 in different Ads and different stages of the disease.

Although substantial progress has been achieved, the study of SIRT1 functions in the immune response is still in the early stage (**Figure 1**). In future studies, experiments are designed to understand how SIRT1 affects different cell types in a coordinated manner within the immune system and the different roles of SIRT1 in different subsets of T cells, B cells, and dendritic cells will be valuable. The precise function of SIRT1 in the development of Ads remains unclear, and future studies are also required to elucidate the molecular pathways and targets regulated by SIRT1 and their roles in treating Ads, which can be used to design precise and more efficient therapies with limited detrimental or unwanted effects. However, new models, methods, and techniques for investigating SIRT1 must be developed to promote its clinical application.

## Author Contributions

PS and XD wrote the first draft of the manuscript. All authors participated in manuscript revision and have approved the submitted version of the manuscript.

## Conflict of Interest

The authors declare that the research was conducted in the absence of any commercial or financial relationships that could be construed as a potential conflict of interest.

## Publisher’s Note

All claims expressed in this article are solely those of the authors and do not necessarily represent those of their affiliated organizations, or those of the publisher, the editors and the reviewers. Any product that may be evaluated in this article, or claim that may be made by its manufacturer, is not guaranteed or endorsed by the publisher.
